# Synthesis‐Related Nanoscale Defects in Mo‐Based Janus Monolayers Revealed by Cross‐Correlated AFM and TERS Imaging

**DOI:** 10.1002/smll.202504742

**Published:** 2025-08-08

**Authors:** Tianyi Zhang, Andrey Krayev, Tilo H. Yang, Nannan Mao, Lauren Hoang, Zhien Wang, Hongwei Liu, Yu‐Ren Peng, Yunyue Zhu, Xudong Zheng, Eleonora Isotta, Maria E. Kira, Ariete Righi, Marcos A. Pimenta, Yu‐Lun Chueh, Eric Pop, Andrew J. Mannix, Jing Kong

**Affiliations:** ^1^ Department of Electrical Engineering and Computer Science Massachusetts Institute of Technology Cambridge MA 02139 USA; ^2^ HORIBA Scientific Novato CA 94949 USA; ^3^ Department of Electrical Engineering Stanford University Stanford CA 94305 USA; ^4^ Department of Materials Science and Engineering National Tsing Hua University Hsinchu 30013 Taiwan; ^5^ Department of Materials Science and Engineering Northwestern University 2220 Campus Drive Evanston IL 60208 USA; ^6^ Departamento de Física Universidade Federal de Minas Gerais Av. Antônio Carlos, 6627, Pampulha Belo Horizonte Minas Gerais 31270‐901 Brazil; ^7^ Department of Materials Science & Engineering Stanford University Stanford CA 94305 USA

**Keywords:** 2D materials, atomic force microscopy, Janus transition metal dichalcogenides, nanoscale defects, tip‐enhanced Raman spectroscopy

## Abstract

2D Janus transition metal dichalcogenides (TMDs) are promising candidates for various applications including non‐linear optics, energy harvesting, and catalysis. These materials are usually synthesized via chemical conversion of pristine TMDs. Nanometer‐scale characterization of the obtained Janus materials’ morphology and local composition is crucial for both the synthesis optimization and the future device applications. In this work, we present the results of cross‐correlated atomic force microscopy (AFM) and tip‐enhanced Raman spectroscopy (TERS) study of Janus monolayers synthesized by the hydrogen plasma‐assisted chemical conversion of MoSe_2_ and MoS_2_. We demonstrate that the choice of both the growth substrate and the starting TMD influences the residual strain, thereby shaping the nanoscale morphology of the resulting Janus material. Furthermore, by employing TERS imaging, we show the presence of nanoscale islands (≈20 nm across) of MoSe_2_‐ ${\mathrm{Mo}}_{\mathrm{Se}}^{S}$ (MoS_2_‐${\mathrm{Mo}}_{S}^{\mathrm{Se}}$) vertical heterostructures originating from the bilayer nanoislands in the precursor monolayer crystals. The understanding of the origins of nanoscale defects in Janus TMDs revealed in this study can help with further optimization of the Janus conversion process towards uniform and wrinkle‐/crack‐free Janus materials. Moreover, this work shows that cross‐correlated AFM and TERS imaging is a powerful and accessible method for studying nanoscale composition and defects in Janus TMD monolayers.

## Graphical abstract

In this work, cross‐correlated atomic force microscopy and tip‐enhanced Raman spectroscopy imaging are demonstrated as a powerful approach to reveal nanoscale composition and defects, such as cracks, wrinkles, and heterostructure islands, in Janus transition metal dichalcogenide monolayers. This provides key insights for synthesis optimization and defect control for potential applications in electronics, optoelectronics, sensing, and catalysis.

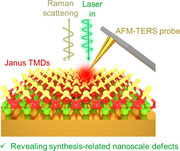

## 1 Introduction

2D Janus transition metal dichalcogenides (TMDs) have an asymmetric structure of X_1_‐M‐X_2_, where a layer of transition metal atoms (denoted as M) is sandwiched between two different chalcogen atom layers (denoted as X_1_ and X_2_).^[^ ^1^ ^]^ Several intriguing properties arise from this unique structure, such as intrinsic vertical dipole and strain anisotropy.^[^ ^2^ ^]^ These emergent properties, coupled with the inherent atomic thickness and semiconducting characteristics, have made Janus TMDs a class of interesting 2D materials for both the studies of their fundamental properties and prospective applications in non‐linear optics, photovoltaics, piezotronics, catalysis, etc.^[^ ^3^ ^]^

Janus TMDs do not exist in the bulk crystal form, neither naturally occurring nor man‐made. Thus, establishing a well‐controlled bottom‐up synthetic route is indispensable for the manufacturing of these monolayers. So far, the synthesis of Janus TMDs has been realized by selectively substituting one of the chalcogen layers in the TMD precursor with a different type of chalcogen. For example, using monolayer MoSe_2_ grown on gold foils as the starting material, the bottom selenium layer at the MoSe_2_‐gold interface was selectively replaced with sulfur at high temperature (≈700 °C), forming Janus ${\mathrm{Mo}}_{S}^{\mathrm{Se}}$ (here Se/S are top/bottom chalcogen layers with respect to the substrate).^[^ ^4^ ^]^ In a different study, a controlled low‐energy selenium implantation method was used to replace the top sulfur layer in monolayer WS_2_, leading to the formation of Janus $W_{S}^{\mathrm{Se}}.$ ^[^ ^5^ ^]^ Additionally, a novel room‐temperature remote hydrogen (H_2_) plasma‐assisted method was developed for the replacement of the top chalcogen layers in TMD crystals grown on SiO_2_/Si, thus realizing a low‐temperature approach for the Janus TMD synthesis.^[^ ^6^ ^]^ Existing reports have demonstrated that the remote H_2_ plasma‐assisted Janus conversion is capable of both converting MS_2_ into Janus $M_{S}^{\mathrm{Se}}$, and converting MSe_2_ into Janus $M_{\mathrm{Se}}^{S}$, seemingly obtaining the same Janus TMD material, but with inversed order of the corresponding chalcogen layers and, consequently, opposite electric dipole orientation.^[^ ^6^, ^7^ ^]^ Nevertheless, the composition, quality, and/or morphology of these two seemingly identical reaction products have not yet been investigated in detail. To achieve this goal, reliable characterization methods with nanometer‐scale spatial resolution are required. Conventional far‐field optical microscopy techniques are rapid approaches to investigate strain, doping, and defects in 2D materials.^[^ ^8^ ^]^ For example, the Janus conversion reaction dynamics, optical quality, and excitonic fine structures have been revealed by either in situ or ex situ Raman and photoluminescence (PL) spectroscopy.^[^ ^9^ ^]^ However, far‐field optical techniques cannot capture fine nanoscale structural features due to the diffraction limit constraint of their spatial resolution. On the other hand, atomic‐resolution characterization approaches, such as scanning transmission electron microscopy (STEM) and scanning tunneling microscopy (STM), can be employed to visualize atomic‐scale defects and local strain in 2D materials.^[^ ^10^ ^]^ Recently, high‐resolution STEM studies have identified several most energetically stable point defects as well as 1D line defects in Janus monolayer $W_{\mathrm{Se}}^{S}$, which helps with correlating the structural defects in Janus materials with their optical features.^[^ ^11^ ^]^ Nevertheless, these approaches are very demanding towards the sample preparation and are not very practical for capturing larger‐area information (50‐1000 nm) on the morphology and uniformity of the investigated materials.^[^ ^12^ ^]^ Moreover, these methods inevitably involve the transfer of Janus TMDs to specific substrates, which still remains rather challenging due to the intrinsic strain within Janus TMD crystals which leads to a strong tendency of forming nanoscrolls, in turn preventing the characterization of the properties of flat Janus crystals.^[^ ^2a^ ^]^

In our current study, we demonstrated cross‐correlated atomic force microscopy (AFM) and tip‐enhanced Raman spectroscopy (TERS) imaging as a powerful and accessible technique to study the nanoscale morphology and chemical composition of Janus ${\mathrm{Mo}}_{\mathrm{Se}}^{S}$ and Janus ${\mathrm{Mo}}_{S}^{\mathrm{Se}}$ monolayers synthesized by H_2_ plasma‐assisted conversion of chemical vapor deposition (CVD)‐grown MoSe_2_ and MoS_2_, respectively. We found that the two conversion pathways induce distinct strain which may lead to the formation of either nanoscale cracks or wrinkles in synthesized Janus TMDs. In addition, TERS characterization is very effective in revealing nanoscale features, such as nano‐islands of MoSe_2_‐Janus ${\mathrm{Mo}}_{\mathrm{Se}}^{S}$ vertical heterostructures of only 15–20 nm across, as well as differentiating the cracks from inverted nano‐wrinkles. Our work provides a solid experimental approach towards the characterization of the nanoscale composition and defects in Janus TMDs, and reveals physical origins of these defects, thus outlining possible approaches towards the synthesis of cracks‐ and wrinkles‐free Janus TMD monolayers.

## 2 Results and Discussion

**Figure 1:**
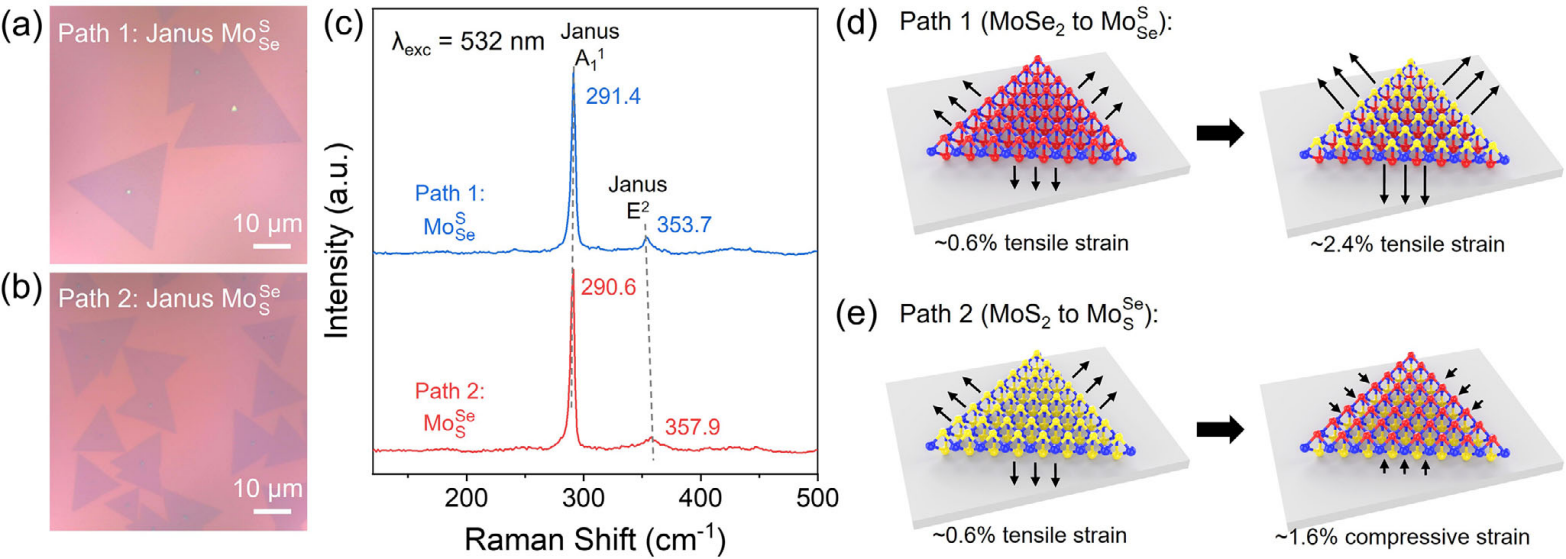
Two reaction pathways of obtaining MoSSe‐type Janus monolayers via either the sulfurization of MoSe_2_ or the selenization of MoS_2_. a,b) Optical microscopy images of as‐synthesized Janus ${\mathrm{Mo}}_{\mathrm{Se}}^{S}$ and ${\mathrm{Mo}}_{S}^{\mathrm{Se}}$ on the SiO_2_/Si substrate. c) Far‐field Raman spectroscopy characterization of as‐grown Janus ${\mathrm{Mo}}_{\mathrm{Se}}^{S}$ and ${\mathrm{Mo}}_{S}^{\mathrm{Se}}$. d,e) Schematics illustrating that the two reaction pathways introduce different types and amount of strain into the resulting Janus monolayers.

The synthesis of MoSSe‐type Janus monolayers can start either from MoSe_2_ or MoS_2_ precursors with consequent sulfurization/selenization of the topmost atomic layer. Using the H_2_ plasma‐assisted atomic‐layer substitution (ALS) method (see Methods), we successfully implemented both reaction pathways, resulting in the ${\mathrm{Mo}}_{\mathrm{Se}}^{S}$ and ${\mathrm{Mo}}_{S}^{\mathrm{Se}}$ monolayers correspondingly (see optical images in **Figure** **1**a,b). At first glance, Janus monolayers obtained via these two routes should be identical, except for their opposite atomic layer order in Z direction. However, it turns out not to be the case, as evidenced by the far‐field Raman spectroscopy characterization using 532 nm excitation (Figure 1c). Although both ${\mathrm{Mo}}_{\mathrm{Se}}^{S}$ and ${\mathrm{Mo}}_{S}^{\mathrm{Se}}$ display characteristic out‐of‐plane A_1_^1^ and in‐plane E^2^ modes associated with Janus MoSSe,^[^ ^13^ ^]^ the frequency of in‐plane E^2^ mode displays a difference of > 4 cm^−1^ between these two types of Janus monolayers. As the in‐plane Raman mode in 2D TMDs is sensitive to in‐plane strain,^[^ ^8a^ ^]^ the observed frequency shift of E^2^ mode suggests a difference in residual strain in Janus ${\mathrm{Mo}}_{\mathrm{Se}}^{S}$ and ${\mathrm{Mo}}_{S}^{\mathrm{Se}}$ crystals. The strain in Janus monolayers is induced during both the TMD growth and the ALS reaction stages, but the degree and even the type of final strain in ${\mathrm{Mo}}_{\mathrm{Se}}^{S}$ and ${\mathrm{Mo}}_{S}^{\mathrm{Se}}$ should differ, because at the ALS reaction stage they experience different lattice expansion/contraction processes. More specifically, as illustrated in Figure 1d,e, based on our theoretical estimates from Raman spectra (see Figure S1, Supporting Information), the monolayer MoS_2_ and MoSe_2_ grown on SiO_2_/Si should acquire a tensile strain of ≈0.6% due to the strong interaction (pinning) between the TMD crystal and the growth substrate,^[^ ^14^ ^]^ and the significant mismatch between the thermal expansion coefficients (TECs) of the TMD and the substrate.^[^ ^15^ ^]^ The ALS process introduces additional strain due to the lattice constant difference between pristine TMDs and the corresponding Janus TMDs. The strain introduced in this process is expressed as: $\varepsilon =\frac{\alpha_{\mathrm{strained}\;\mathrm{Janus}}\;-\;\alpha_{\mathrm{relaxed}\;\mathrm{Janus}}}{\alpha_{\mathrm{relaxed}\;\mathrm{Janus}}}\;.$ Here α_relaxed Janus_ and α_strained Janus_ represent the lattice constants of Janus MoSSe without and with strain, respectively. During the ALS process, since the 2D crystal is strongly pinned to the substrate, the as‐synthesized Janus TMDs should be forced to adopt the same lattice constant with the starting TMDs (i.e., α_strained Janus_ = α_TMDs_ ). Thus, the equation for strain calculation can be rewritten as: $\varepsilon =\frac{\alpha_{\mathrm{TMDs}}\;-\;\alpha_{MoSSe}}{\alpha_{MoSSe}}$. It enables us to use literature reported lattice constant values to obtain the strain estimation.^[^ ^2^, ^16^ ^]^

In the case of converting MoSe_2_ to ${\mathrm{Mo}}_{\mathrm{Se}}^{S}$, pre‐existing tensile strain in CVD‐grown MoSe_2_ is augmented by additional ≈1.8% tensile strain caused by sulfurization of the top layer (the lattice constant is decreased from $\alpha_{\mathrm{MoS}e_{2}}=3.288\;\;Å$ to α_MoSSe_ =  3.23 Å), leading to the final tensile strain of ≈2.4% (Figure 1d). In contrast, upon conversion of MoS_2_ to ${\mathrm{Mo}}_{S}^{\mathrm{Se}}$, due to the replacement of the top sulfur atomic layer with selenium, a ≈2.2% compressive strain should appear (the lattice constant is increased from $\alpha_{\mathrm{Mo}S_{2}}=3.16\;\;Å$ to α_MoSSe_ =  3.23 Å), but this compressive strain is partially compensated by the original tensile strain, leading to residual compressive strain value in ${\mathrm{Mo}}_{S}^{\mathrm{Se}}$ crystals of ≈1.6% (Figure 1e). We would like to stress again that the seemingly counterintuitive sign of strain in response to the lattice constant increase/decrease at the conversion stage is caused by the strong pinning of the TMD and later‐ Janus TMD crystals to the substrate.

Having understood the in‐plane strain difference in Janus monolayer ${\mathrm{Mo}}_{\mathrm{Se}}^{S}$ and ${\mathrm{Mo}}_{S}^{\mathrm{Se}}$ converted from MoSe_2_ and MoS_2_, respectively, we can now focus on the comparison of the corresponding crystal morphologies. Janus TMD crystals were assessed by cross‐correlated AFM and TERS imaging (see Experimental section for details on the synthesis of Janus TMDs) on both the growth substrates and after a gold‐ or silver‐assisted transfer. The latter transfer from the growth substrate (SiO_2_/Si or fused silica) was performed in order to maximize TERS response by enabling the so‐called gap mode TERS conditions. Here we followed the previously developed metal‐assisted dry‐transfer procedure (Figure S2a, Supporting Information).^[^ ^17^ ^]^ This transfer process results in Janus TMDs embedded in the gold or silver substrate, which, as mentioned already, enables the optimal gap‐mode conditions for TERS characterization, as well as the efficient use of Kelvin probe microscopy and photocurrent imaging techniques. It is worth noting that this transfer process involves the mechanical delamination and flipping of Janus TMDs, thus exposing the side of the crystal that was originally facing the growth substrate. The schematics of Janus ${\mathrm{Mo}}_{\mathrm{Se}}^{S}$ monolayer and the ${\mathrm{Mo}}_{\mathrm{Se}}^{S}$‐MoSe_2_ heterobilayer before and after transfer are illustrated in Figure S2 (Supporting Information).

**Figure 2:**
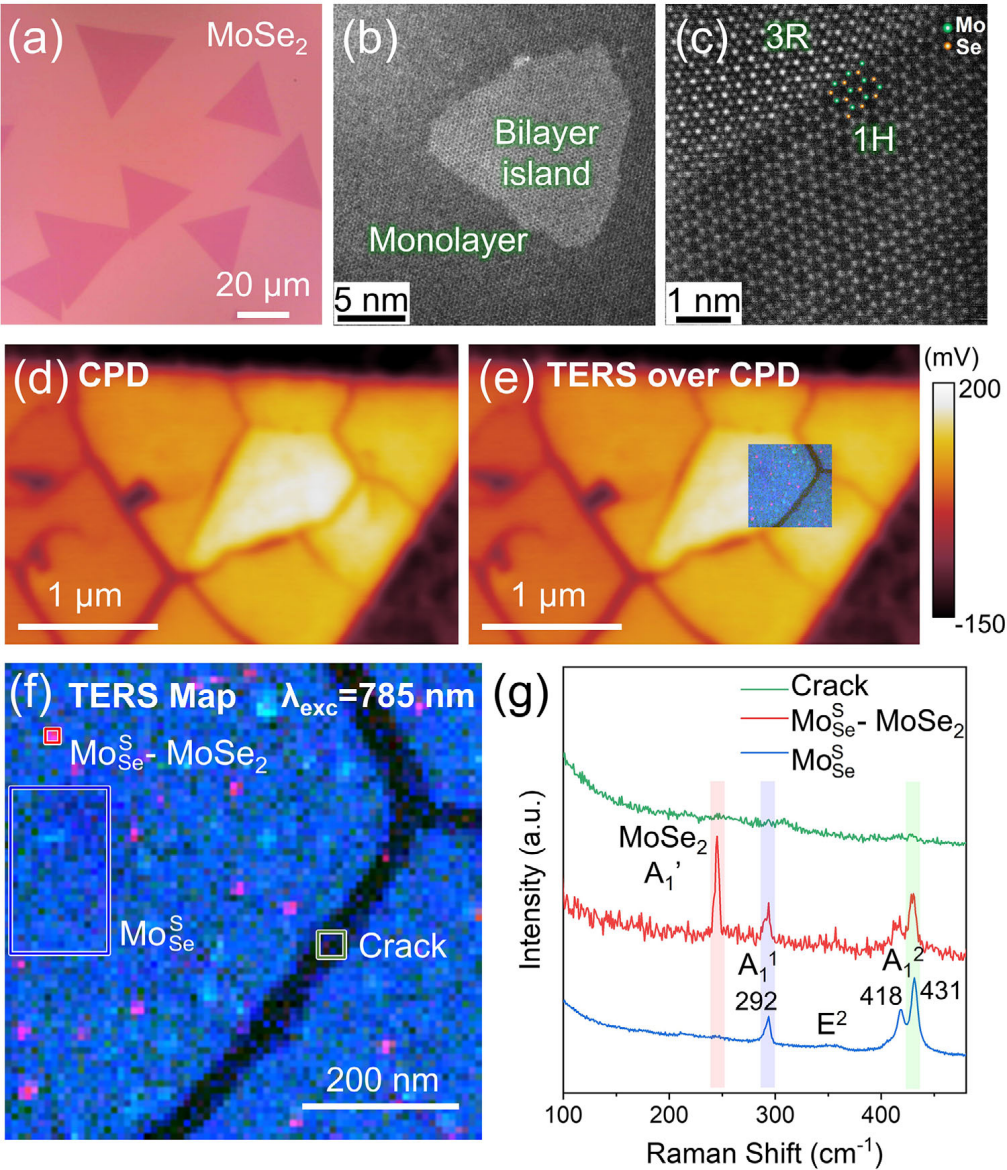
AFM and TERS characterization of Janus ${\mathrm{Mo}}_{\mathrm{Se}}^{S}$ converted from MoSe_2_. a) Optical microscopy image of CVD‐grown MoSe_2_ on SiO_2_/Si which is used as the precursor for the ALS conversion. b) Low‐magnification and c) atomic‐resolution STEM‐HAADF images of monolayer and bilayer regions of CVD‐grown MoSe_2_. d) Contact potential difference (CPD) image of the transferred Janus ${\mathrm{Mo}}_{\mathrm{Se}}^{S}$ flake on the gold substrate. e) The TERS map overlaid over the CPD image of Janus ${\mathrm{Mo}}_{\mathrm{Se}}^{S}$. f) The TERS map showing the intensity distribution of correspondingly highlighted Raman modes in panel (g), namely–MoSe_2_ A_1_’ (red), ${\mathrm{Mo}}_{\mathrm{Se}}^{S}$ A_1_^2^ (green), and ${\mathrm{Mo}}_{\mathrm{Se}}^{S}$ A_1_^1^ (blue). g) The TERS spectra averaged over the correspondingly colored boxes in panel (f), which represent Janus monolayer ${\mathrm{Mo}}_{\mathrm{Se}}^{S}$ (blue), ${\mathrm{Mo}}_{\mathrm{Se}}^{S}$‐MoSe_2_ vertical heterostructures (red), and crack (green) regions.

We first start with the results obtained on ${\mathrm{Mo}}_{\mathrm{Se}}^{S}$ converted from MoSe_2_. The as‐grown MoSe_2_ flakes were primarily triangular monolayer crystals with lateral sizes of 20 µm and more (**Figure** **2**a). From scanning transmission electron microscopy high angle annular dark field (STEM‐HAADF) characterization, we observed the presence of nanoscale bilayer MoSe_2_ islands with lateral sizes of ≈20 nm (Figure 2b), and the majority of these islands were 3R‐stacked (rhombohedral),^[^ ^18^ ^]^ as confirmed by the atomic‐resolution STEM‐HAADF image in Figure 2c. Upon conversion to Janus ${\mathrm{Mo}}_{\mathrm{Se}}^{S}$, the resulting crystals fragmented into smaller, approximately 500–1000 nm‐across, physically separated domains due to excessive tensile strain introduced into the lattice, as evidenced by the AFM topography characterization (Figure S3, Supporting Information) as well as surface potential and TERS characterization that will be discussed in the following part of the manuscript.

We performed Kelvin probe microscopy and TERS imaging (785 nm excitation) of Janus ${\mathrm{Mo}}_{\mathrm{Se}}^{S}$ transferred to gold. Contact potential difference (CPD) images in Figure 2d clearly show the presence of small domains, which are similar in size and shape to the ones observed in the AFM topography of as‐synthesized Janus ${\mathrm{Mo}}_{\mathrm{Se}}^{S}$ crystals (Figure S3, Supporting Information). A TERS map was further collected over the junction of two adjacent domains (Figure 2e,f) to reveal detailed chemical composition information on both the domains and the junction lines. The red, green, and blue colors in the combined TERS map (Figure 2f) represent the intensities of A_1_’(MoSe_2_), A_1_^2^(${\mathrm{Mo}}_{\mathrm{Se}}^{S})$, and A_1_^1^(${\mathrm{Mo}}_{\mathrm{Se}}^{S})$ vibrational modes, respectively. From the representative averaged TERS spectra (blue‐colored spectrum in Figure 2g) collected over the main body of the domain (marked with the blue rectangle in Figure 2f), we see a clear signature of Janus monolayer ${\mathrm{Mo}}_{\mathrm{Se}}^{S}$ with characteristic A_1_^1^ mode at ≈292 cm^−1^ and A_1_^2^ at ≈431 cm^−1^. The Raman modes of the starting MoSe_2_ are absent, indicating a full conversion to Janus ${\mathrm{Mo}}_{\mathrm{Se}}^{S}$. Interestingly, the ${\mathrm{Mo}}_{\mathrm{Se}}^{S}$ A_1_^2^ band is more intense than A_1_^1^, and is also noticeably split. We observed no characteristic Raman modes in the cracks between the adjacent domains (green‐colored spectrum in Figure 2g), which confirms that these domains are physically separated with ≈30 nm wide gaps. We note that the crack formation in Janus ${\mathrm{Mo}}_{\mathrm{Se}}^{S}$ monolayers is reproducible across different synthesis batches, as demonstrated by an additional scanning electron microscopy (SEM) image from a separate batch of synthesized Janus ${\mathrm{Mo}}_{\mathrm{Se}}^{S}$ (Figure S4a, Supporting Information). This suggests that the crack formation is due to intrinsic strain‐related factors rather than experimental variations. Additionally, to examine whether similar strain‐induced cracking occurs in Janus TMDs based on other transition metals (e.g., W‐based), we also used SEM to characterize the morphology of Janus $W_{\mathrm{Se}}^{S}$ converted from WSe_2_ (Figure S4b, Supporting Information). It is found that as‐grown Janus $W_{\mathrm{Se}}^{S}$ monolayers exhibit fewer cracks compared to Janus ${\mathrm{Mo}}_{\mathrm{Se}}^{S}$, and those cracks are mainly formed near the flake edges. The reason may be related to the differences in the fracture strengths or the amount of Janus conversion‐induced strain in our Janus ${\mathrm{Mo}}_{\mathrm{Se}}^{S}$ and $W_{\mathrm{Se}}^{S}$, which warrants future investigations.

Finally, we found small islands with sizes of 20–30 nm (marked with the red square in Figure 2f) which showed simultaneously strong intensity of A_1_’ mode of MoSe_2_ at ≈242 cm^−1^ and the typical Raman modes of Janus ${\mathrm{Mo}}_{\mathrm{Se}}^{S}$, which is a spectroscopic signature of the Janus ${\mathrm{Mo}}_{\mathrm{Se}}^{S}$‐MoSe_2_ vertical heterostructures (schematically shown in Figure S2c, Supporting Information). This correlates very well with the STEM observation of the bilayer MoSe_2_ nano‐islands in the precursor material, as the self‐limiting ALS method converts only the topmost Se layer into S, resulting in formation of ${\mathrm{Mo}}_{\mathrm{Se}}^{S}$ only in the top layer of the bilayer MoSe_2_. It should be noted again that conventional far‐field Raman microscopy of the as‐grown Janus TMDs is not able to resolve these nanoscale heterostructures or cracks due to insufficient spatial resolution (Figure S5, Supporting Information). This further highlights the immense potential of using TERS imaging to capture detailed morphological and chemical information of Janus TMDs with relevant, nm‐scale spatial resolution.

To further confirm the multi‐layer nature of the nano‐islands in Janus ${\mathrm{Mo}}_{\mathrm{Se}}^{S}$ crystals, we collected another TERS map on an adjacent domain. In this domain, we recorded the photocurrent generated across the crystal (along Z direction, between the TERS probe and the substrate) together with the TERS spectra in each pixel of the map (Figure S6, Supporting Information). Comparing the TERS and photocurrent maps in Figure S6e,f (Supporting Information), we can see that the photocurrent greatly increases over the nanoislands that show spectral signatures of both the Janus ${\mathrm{Mo}}_{\mathrm{Se}}^{S}$ and starting MoSe_2_. Enhanced photocurrent should be expected in the vertical heterostructures, because for 2D TMDs with a few‐layer thicknesses, the photocurrent value is limited by the absorption in the material which grows with the number of layers, at least within the low layer number limit. The exact scaling of the photocurrent with the layer number of the TMD crystals may vary depending on the excitation wavelength, showing almost an exponential growth under illumination slightly below the A exciton energy in the case of few‐layer WS_2_ (Figure S7, Supporting Information).

**Figure 3:**
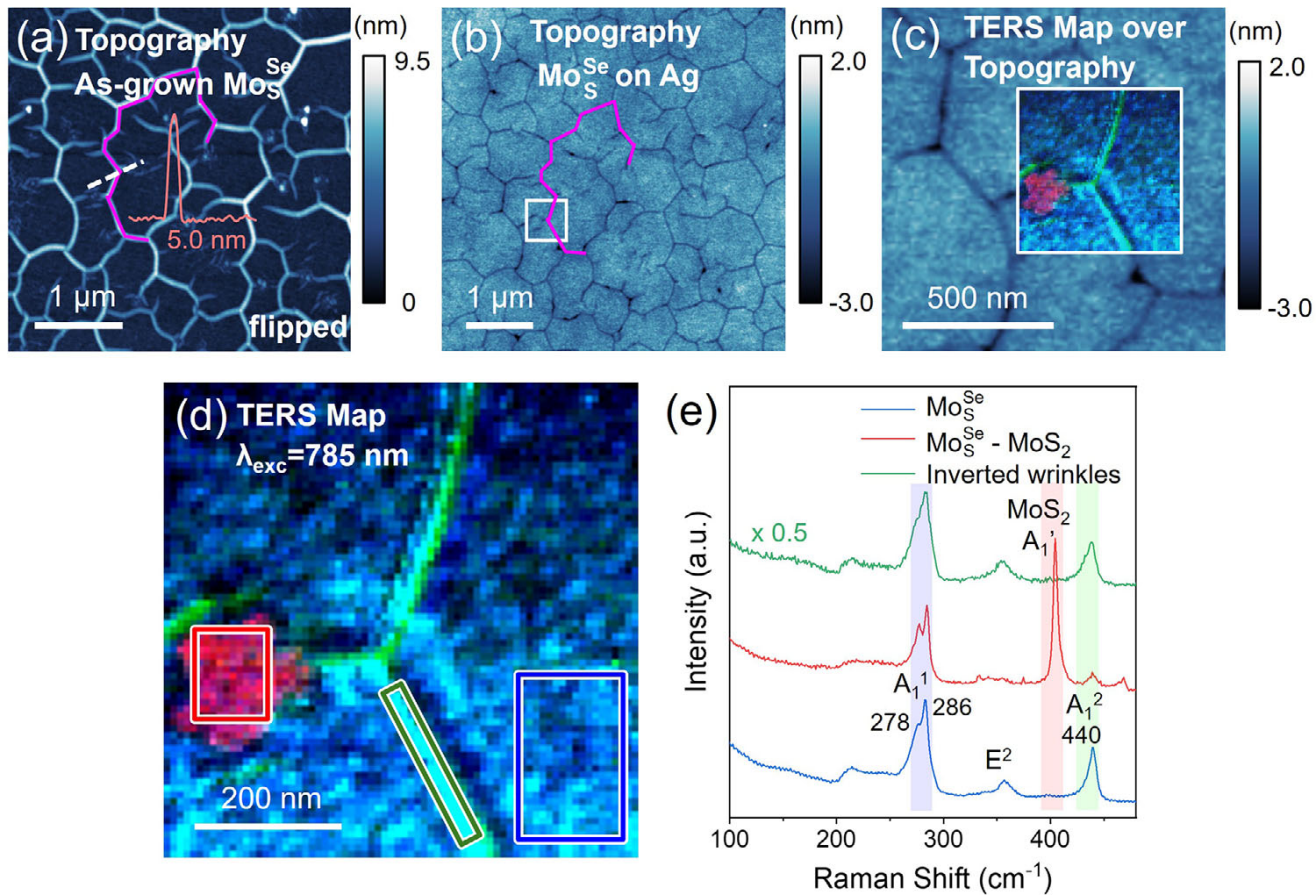
AFM and TERS characterization of Janus ${\mathrm{Mo}}_{S}^{\mathrm{Se}}$ converted from MoS_2_. a) AFM topography image (image flipped to compare the mirror inversion after the transfer) of as‐grown Janus ${\mathrm{Mo}}_{S}^{\mathrm{Se}}$ on SiO_2_/Si. Inset: an AFM line profile of a typical wrinkle in Janus ${\mathrm{Mo}}_{S}^{\mathrm{Se}}$, showing a height of 5.0 nm. b) AFM topography image of the same region of the Janus ${\mathrm{Mo}}_{S}^{\mathrm{Se}}$ crystal after being transferred onto silver. c) Magnified AFM topography image of the region highlighted by the white square in (b). The TERS map is also acquired from this region, and is superimposed on the topography image. d) The magnified TERS map showing the intensity distribution of correspondingly highlighted Raman modes in panel (e), namely–MoS_2_ A_1_’ (red), ${\mathrm{Mo}}_{S}^{\mathrm{Se}}$ A_1_^2^ (green), and ${\mathrm{Mo}}_{S}^{\mathrm{Se}}$ A_1_^1^ (blue). e) The TERS spectra averaged over the correspondingly colored boxes in panel (d), which represent Janus monolayer ${\mathrm{Mo}}_{S}^{\mathrm{Se}}$ (blue), ${\mathrm{Mo}}_{S}^{\mathrm{Se}}$‐MoS_2_ vertical heterostructures (red), and inverted wrinkle (green) regions.

Now we switch to the results obtained on Janus ${\mathrm{Mo}}_{S}^{\mathrm{Se}}$ crystals converted from MoS_2_. In order to understand the morphological features of Janus ${\mathrm{Mo}}_{S}^{\mathrm{Se}}$, we first collected the AFM topography image of a specific crystal on the growth substrate (**Figure** **3**a) and then located the same crystal after the silver‐assisted transfer to perform another AFM characterization (Figure 3b). We confirmed that the two AFM images were obtained on the exact same area by superimposing the before‐ (the image is flipped to properly represent the mirror inversion after the transfer) and after‐the‐transfer topography (Figure S8, Supporting Information), that produced a perfect match. From the topography image in Figure 3a, wrinkles with typical heights of ≈5 nm are observed on as‐grown Janus ${\mathrm{Mo}}_{S}^{\mathrm{Se}}$. We assign their appearance to the compressive strain introduced into the lattice during the Janus conversion of MoS_2_, as we discussed earlier (Figure 1 and related discussion). Our observations confirm that the different types and the amount of strain induced during the ALS conversion stage can lead to fundamentally different morphologies in resulting Janus TMDs.

Though the wrinkles got inverted upon the silver‐assisted transfer process and looked like the cracks in Janus ${\mathrm{Mo}}_{\mathrm{Se}}^{S}$ crystals (Figure 3b), TERS imaging (785 nm excitation) clearly differentiated these inverted wrinkles from the cracks. Figure 3c presents a magnified AFM topography image of the region highlighted by the white square in Figure 3b. A TERS map was collected from the white square‐marked region in Figure 3c, and is shown in Figure 3d. Similar to the TERS image of Janus ${\mathrm{Mo}}_{\mathrm{Se}}^{S}$ discussed earlier, the colors in TERS map in Figure 3d represent the intensities of A_1_’(MoS_2_)‐red, A_1_^2^(${\mathrm{Mo}}_{S}^{\mathrm{Se}}$)‐green, and A_1_^1^(${\mathrm{Mo}}_{S}^{\mathrm{Se}}$)‐blue modes, correspondingly. From both the TERS map (regions marked with the green rectangle) and the individual TERS spectra (Figure 3e), we can clearly see that the TERS signal of Janus ${\mathrm{Mo}}_{S}^{\mathrm{Se}}$ enhances over the inverted wrinkles instead of vanishing, confirming the presence of wrinkles rather than cracks. We also observed islands of Janus ${\mathrm{Mo}}_{S}^{\mathrm{Se}}$‐MoS_2_ vertical heterostructures (≈100 nm across, as marked with the red rectangle in Figure 3d), which is evidenced by the strong A_1_’ mode of MoS_2_ at ≈405 cm^−1^ and the typical Raman modes of Janus ${\mathrm{Mo}}_{S}^{\mathrm{Se}}$. Notably, while the microscopic islands of vertical heterostructures of Janus ${\mathrm{Mo}}_{S}^{\mathrm{Se}}$‐MoS_2_ were captured both by the Kelvin probe microscopy and TERS imaging, the nanoscale islands were only detected by TERS imaging (Figure S9, Supporting Information), which again highlights the power of this technique for the nanoscale characterization of Janus TMDs and other 2D semiconductors. The reason for such seemingly counterintuitive discrepancy between the spatial resolution of the Kelvin probe and TERS maps is related to the fact that in Kelvin measurements the spatial resolution is limited by the tip radius (50–100 nm for TERS probes) and the tip‐sample separation in the Kelvin pass (20–30 nm), while in TERS measurements performed de facto in contact mode, it is the thickness of the TMD layer (≈1‐2 nm) that separates the tip and the substrate which is the scaling factor for the spatial resolution.

Additionally, we noticed significant differences in TERS spectra of Janus monolayer ${\mathrm{Mo}}_{S}^{\mathrm{Se}}$ with ${\mathrm{Mo}}_{\mathrm{Se}}^{S}$ crystals. Specifically, in ${\mathrm{Mo}}_{S}^{\mathrm{Se}}$, the A_1_^1^ mode is more intense than A_1_^2^ (blue‐colored spectra in Figure 3e), which is the opposite of what we observed in ${\mathrm{Mo}}_{\mathrm{Se}}^{S}$ (blue‐colored spectra in Figure 2g). Moreover, A_1_^1^ is obviously split in Janus ${\mathrm{Mo}}_{S}^{\mathrm{Se}}$, when compared to the case of the A_1_^2^ peak splitting in the TERS spectrum of Janus ${\mathrm{Mo}}_{\mathrm{Se}}^{S}$. The possible mechanism that leads to these differences will be discussed later in the manuscript.

While the wrinkle formation within the Janus TMD crystals may open up their potential applications in sensing and catalysis, the ability to yield Janus TMDs with a flat and uniform surface morphology is desirable for prospective electronic and photonic applications. To attain this goal, our rationale is to reduce the amount of compressive strain in the synthesized Janus ${\mathrm{Mo}}_{S}^{\mathrm{Se}}$ by substrate engineering to mitigate wrinkle formation. In Figure 1d,e, the amount of strain induced during the TMD synthesis process is tunable by selecting substrates with different TECs,^[^ ^19^ ^]^ while the strain induced during the MoS_2_ → Janus ${\mathrm{Mo}}_{S}^{\mathrm{Se}}$ conversion process is intrinsically determined by their lattice constant differences. Thus, we focused on using alternative substrates other than SiO_2_/Si to introduce a larger amount of tensile strain in the precursor MoS_2_, which can better compensate for the compressive strain during the Janus conversion process, decreasing the amount of remaining strain in Janus ${\mathrm{Mo}}_{S}^{\mathrm{Se}}$. To enhance the tensile strain in CVD‐grown MoS_2_, a low TEC substrate is desirable.^[^ ^19^ ^]^ Here, we selected fused silica with a very low TEC value of 0.55 × 10^−6^ K^−1^ as a suitable substrate. In comparison, the TEC of SiO_2_/Si is expected to be governed by the TEC of Si (i.e., 2.7 × 10^−6^ K^−1^ at room temperature),^[^ ^20^ ^]^ due to the much smaller thickness of the SiO_2_ layer. Thus, a larger TEC mismatch between fused silica and MoS_2_ (measured to be 7.6 ± 0.9 × 10^−6^ K^−1^)^[^ ^21^ ^]^ can lead to increased tensile strain in CVD‐grown MoS_2_. In CVD‐synthesized monolayer MoS_2_ on fused silica, we observed red shifts in the MoS_2_ E’ Raman mode and PL emission compared to MoS_2_ synthesized on SiO_2_/Si (Figure S10, Supporting Information). This further confirms the presence of increased tensile strain in MoS_2_, as tensile strain leads to phonon softening and bandgap reduction, which induces red shifts in both Raman E’ mode and PL emission energy.^[^ ^15^, ^22^ ^]^

**Figure 4:**
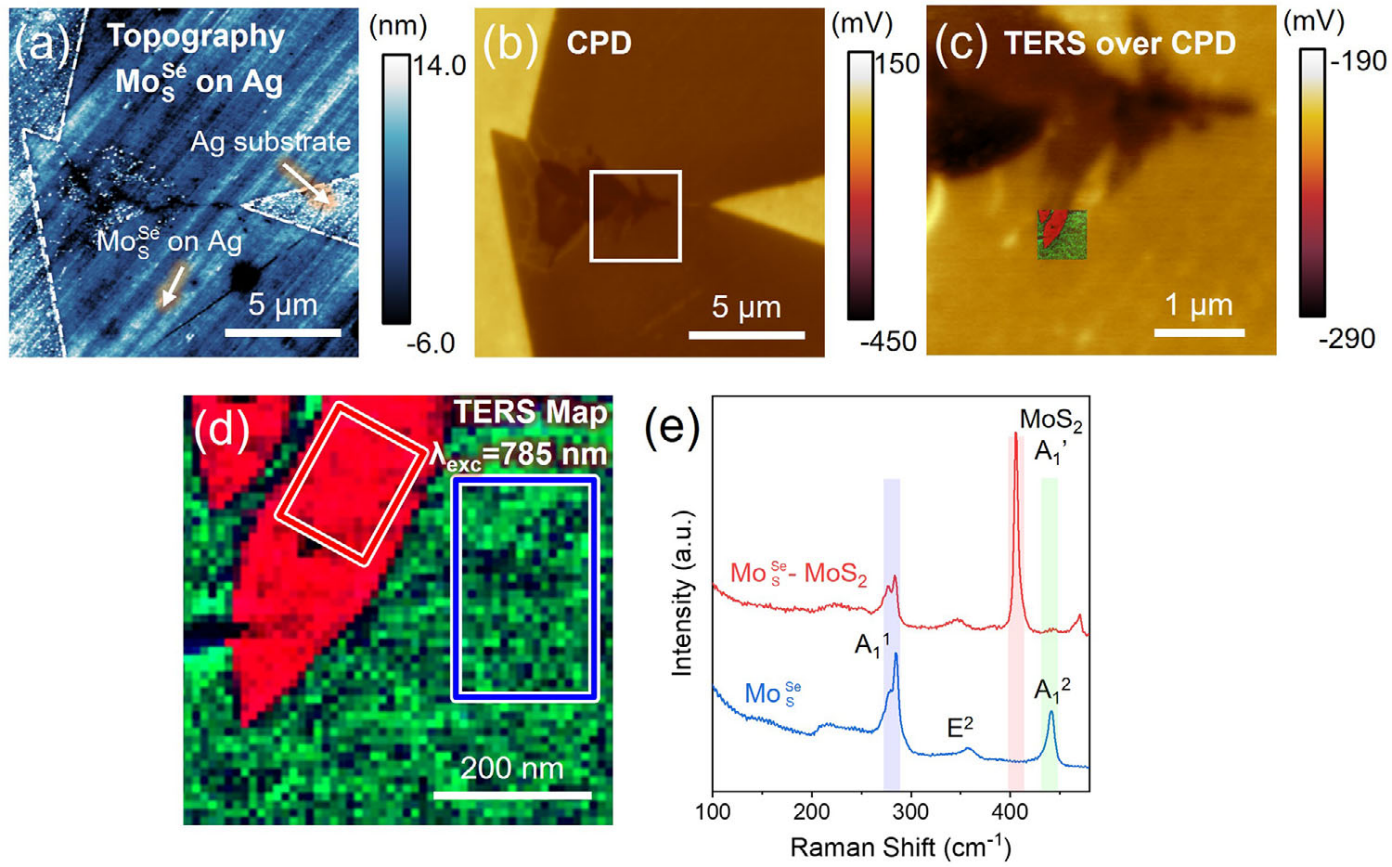
AFM and TERS characterization of wrinkle‐free Janus ${\mathrm{Mo}}_{S}^{\mathrm{Se}}$ synthesized on the fused silica substrate. a) AFM topography image of Janus ${\mathrm{Mo}}_{S}^{\mathrm{Se}}$ transferred from fused silica to silver. The edges of ${\mathrm{Mo}}_{S}^{\mathrm{Se}}$ are outlined by white dashed lines to differentiate ${\mathrm{Mo}}_{S}^{\mathrm{Se}}$ and bare silver substrate regions. b) CPD image of the identical Janus ${\mathrm{Mo}}_{S}^{\mathrm{Se}}$ flake. c) Magnified CPD image of the region highlighted by the white square in (b). The TERS map is also acquired from this region, and is superimposed on the CPD image. d) The magnified TERS map showing the intensity distribution of correspondingly highlighted Raman modes in panel (e), namely–MoS_2_ A_1_’ (red), ${\mathrm{Mo}}_{S}^{\mathrm{Se}}$ A_1_^2^ (green), and ${\mathrm{Mo}}_{S}^{\mathrm{Se}}$ A_1_^1^ (blue). e) The TERS spectra averaged over the correspondingly colored boxes in panel (d), which represent Janus monolayer ${\mathrm{Mo}}_{S}^{\mathrm{Se}}$ (blue) and ${\mathrm{Mo}}_{S}^{\mathrm{Se}}$‐MoS_2_ vertical heterostructures (red).

Similar to the case of Si/SiO_2_ substrates, we synthesized Janus ${\mathrm{Mo}}_{S}^{\mathrm{Se}}$ on fused silica and characterized the same flake both as‐grown and after the silver‐assisted transfer (**Figures** **4** and S11, Supporting Information). Note that we chose silver as the transfer medium and TERS substrate in order to extend the spectral range of TERS measurements to 473 nm, while still preserving TERS enhancement in NIR optical range.^[^ ^23^ ^]^ From the topography image of as‐synthesized ${\mathrm{Mo}}_{S}^{\mathrm{Se}}$ sample grown on fused silica in Figure S11 (Supporting Information), the major part of the crystal has very smooth topography, indicating that the use of fused silica substrate mitigates the wrinkle formation. A small dendrite‐like region is also present in the crystal, which shows a surface potential difference in the CPD map (Figure 4b). TERS measurements under 785 nm excitation confirmed that this region is the vertical heterostructure of Janus ${\mathrm{Mo}}_{S}^{\mathrm{Se}}$‐MoS_2_ (Figure 4d,e). In terms of the TERS spectra from both the monolayer Janus ${\mathrm{Mo}}_{S}^{\mathrm{Se}}$ and Janus ${\mathrm{Mo}}_{S}^{\mathrm{Se}}$‐MoS_2_ vertical heterostructures (Figure 4e), they appeared to be very similar to the spectra from the corresponding crystals grown on SiO_2_/Si (Figure 3e), indicating a comparable quality of Janus ${\mathrm{Mo}}_{S}^{\mathrm{Se}}$ and vertical heterostructures grown on fused silica and Si/SiO_2_.

**Figure 5:**
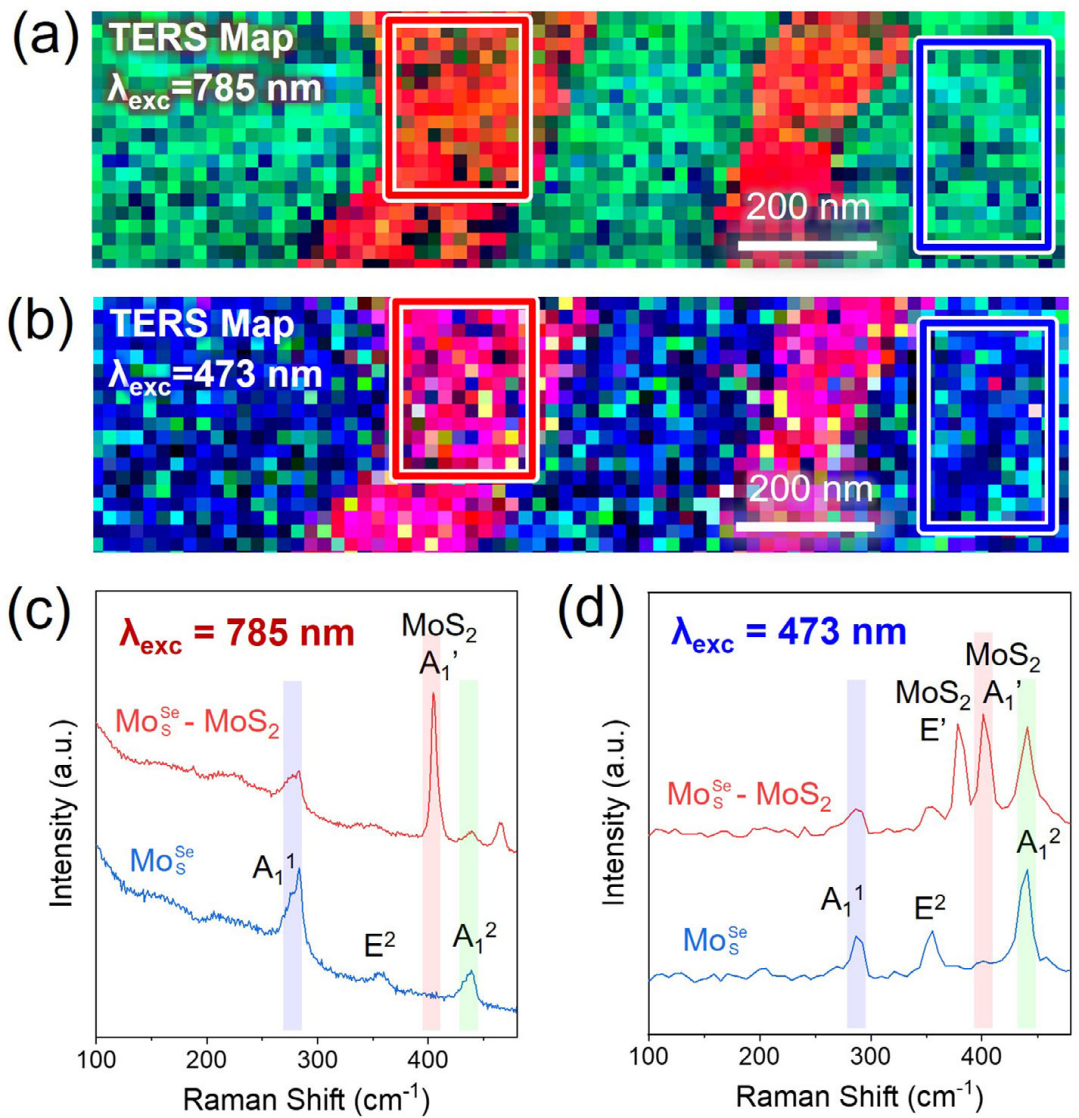
Excitation‐dependent TERS characterization of Janus ${\mathrm{Mo}}_{S}^{\mathrm{Se}}$ on silver. a,b) TERS maps of an identical region of Janus ${\mathrm{Mo}}_{S}^{\mathrm{Se}}$ using 785 nm and 473 nm excitation, respectively. The color of TERS maps indicates the intensity distribution of correspondingly highlighted Raman modes in panels (c,d), namely– MoS_2_ A_1_’ (red), ${\mathrm{Mo}}_{S}^{\mathrm{Se}}$ A_1_^2^ (green), and ${\mathrm{Mo}}_{S}^{\mathrm{Se}}$ A_1_^1^ (blue). c,d) The TERS spectra averaged over the correspondingly colored boxes in panels (a,b), which represent Janus monolayer ${\mathrm{Mo}}_{S}^{\mathrm{Se}}$ (blue) and ${\mathrm{Mo}}_{S}^{\mathrm{Se}}$‐MoS_2_ vertical heterostructures (red).

Finally, we attempted to gain a better understanding towards the nature of spectral splitting of A_1_^1^ and A_1_^2^ modes in Janus ${\mathrm{Mo}}_{S}^{\mathrm{Se}}$ and Janus ${\mathrm{Mo}}_{\mathrm{Se}}^{S}$ monolayers by excitation‐dependent TERS characterization. We collected TERS maps of the same area in the Janus ${\mathrm{Mo}}_{S}^{\mathrm{Se}}$ sample on silver with the same TERS probe at 785 and 473 nm excitation. The TERS maps are presented in **Figure** **5**a,b, and the individual spectra are extracted from the regions marked with blue and red rectangles, which correspond to monolayer Janus ${\mathrm{Mo}}_{S}^{\mathrm{Se}}$ and Janus ${\mathrm{Mo}}_{S}^{\mathrm{Se}}$‐MoS_2_ vertical heterostructures, respectively (Figure 5c,d). From the TERS spectra corresponding to monolayer Janus ${\mathrm{Mo}}_{S}^{\mathrm{Se}}$, we notice a significant increase of the relative intensity of A_1_^2^ mode compared to the A_1_^1^ in the spectrum collected with 473 nm excitation (Figure 5d). The difference in TERS spectra obtained using 785 nm and 473 nm excitations is likely due to the excitation laser wavelength dependence of Raman intensities, which should be valid for both the far‐field Raman (Figure S12, Supporting Information) and TERS.^[^ ^13^ ^]^ In addition, the obvious split of the A_1_^1^ mode observed in TERS spectra collected with 785 nm excitation is not visible in TERS spectra of the same area collected with 473 nm, though it may also be a consequence of insufficient spectral resolution in the latter case. We should also note that in the case of 473 nm excitation, we observe both the out‐of‐plane A_1_’ and the in‐plane E’ modes of MoS_2_ in Janus ${\mathrm{Mo}}_{S}^{\mathrm{Se}}$‐MoS_2_ vertical heterostructures marked with red rectangles in TERS maps in Figure 5b, while the spectrum collected with 785 nm features only A_1_’ mode. The appearance/absence of Raman bands in 2D TMDs with varied excitation reflects various electronic resonances and intricate electron‐phonon interactions in these materials.^[^ ^24^ ^]^ We speculate that the observed A_1_^1^ and A_1_^2^ splitting observed in Janus ${\mathrm{Mo}}_{S}^{\mathrm{Se}}$ and ${\mathrm{Mo}}_{\mathrm{Se}}^{S}$ monolayers may also be a consequence of resonant conditions that can lead to the rise of some bands at certain excitation which are not visible at different excitation wavelengths. More specifically, the distinct strain and/or potentially different defect concentrations in our Janus ${\mathrm{Mo}}_{S}^{\mathrm{Se}}$ and ${\mathrm{Mo}}_{\mathrm{Se}}^{S}$ are likely to modify their electronic band structures, which can lead to different excitation‐energy dependence of individual Raman bands. We must state though that a firm, well‐experimentally supported answer to the question of the nature of the A_1_^1^ and A_1_^2^ mode splitting as well as the obvious inversion of the intensity of these bands in ${\mathrm{Mo}}_{S}^{\mathrm{Se}}$ and ${\mathrm{Mo}}_{\mathrm{Se}}^{S}$ crystals require a focused dedicated study, which is beyond the scope of our current manuscript.

## 3 Conclusion

In this work, using cross‐correlated AFM and TERS imaging, we investigated the typical synthesis‐related nanoscale defects in Janus ${\mathrm{Mo}}_{\mathrm{Se}}^{S}$ and ${\mathrm{Mo}}_{S}^{\mathrm{Se}}$ monolayers. We identified two principal types of defects—one originating from the presence of nano‐ to micro‐scale multilayer islands in the predominantly monolayer TMD precursor crystals. Upon conversion, these islands become vertical heterostructures of the Janus material and the corresponding starting TMD underneath. The second type of defect is related to the type and the amount of residual strain incurred in crystals in the course of the TMD synthesis and Janus conversion. In the case of MoSe_2_, the synthesis‐induced tensile strain is augmented by the additional tensile strain incurred during the Janus conversion which shatters Janus ${\;\mathrm{Mo}}_{\mathrm{Se}}^{S}$ crystals into small, 500–1000 nm across domains separated by physically few‐nanometers‐wide gaps. In contrast, when using MoS_2_ as the precursor, the original tensile strain is compensated by the compressive strain upon Janus conversion which may lead to the formation of nano‐wrinkles, with preserved continuity of Janus material. We demonstrated that the amount of the remaining compressive strain in ${\mathrm{Mo}}_{S}^{\mathrm{Se}}$, and consequently its topography, may be controlled by the choice of the original growth substrate. By substituting SiO_2_/Si substrate with fused silica, we reduced the residual compressive strain in Janus ${\mathrm{Mo}}_{S}^{\mathrm{Se}}$ and successfully obtained almost wrinkle‐free Janus ${\mathrm{Mo}}_{S}^{\mathrm{Se}}$ crystals.

We hope that the information obtained in our study demonstrates the power of the cross‐correlated AFM and TERS imaging of 2D materials and will be helpful for further optimization of the Janus conversion process. This would allow consistent synthesis of Janus monolayers free of cracks, bilayers, and wrinkles, as well as the precise control over strain and defects in these materials for potential applications such as optoelectronics, sensing, and catalysis. Moreover, we envision that the capabilities of AFM and TERS imaging can be further enhanced by integrating in situ strain setups and/or tip‐enhanced photoluminescence (TEPL) techniques, which will offer valuable insights into strain‐modulated phenomena in 2D materials, such as defect evolution, lattice reconfigurations, and light‐matter interactions.

## 4 Experimental Section

### AFM and TERS Measurements

AFM and TERS were conducted on a LabRam‐Nano AFM‐Raman system (HORIBA Scientific) equipped with 7 excitation lasers at 830, 785, 671, 633, 594, 532 and 473 nm. Excitation and collection of TERS signal was done using the side 100×, 0.7 NA objective (Mitutoyo) inclined at 25° to the sample plane with *p* polarization of the excitation laser. Gold‐coated or the Type II protected silver probes (HORIBA Scientific) based on Access‐SNC AFM cantilevers (APPNano) were used for both the SPM and TERS characterization. Laser power past the objective for individual wavelengths was kept at the level of 100–250 µW. The colors in combined TERS maps that were representing the intensities of different Raman bands were adjusted individually for red, blue and green components without any strict protocol (i.e., red, blue, and green channels have their individual scale bars) in such a way that the structural features observed in corresponding combined maps were most clearly highlighted.

### Synthesis of Janus TMDs

The starting pristine TMDs (MoS_2_ and MoSe_2_) were first grown on SiO_2_ (300 nm)/Si and double‐side polished fused silica substrates by CVD. The molybdenum precursor solution was prepared by mixing molybdenum trioxide (MoO_3_) and potassium iodide (KI) in ammonia (NH_4_OH), following a previously reported approach.^[^ ^25^ ^]^ Subsequently, the solution was spin‐coated onto substrates, and placed inside a one‐inch tube furnace for sulfurization/selenization. The syntheses of MoS_2_ and MoSe_2_ were carried out at 750 °C and 850 °C for 5 min, respectively. For the MoS_2_ synthesis, pure Ar (20 sccm) was applied as the carrier gas. In comparison, the MoSe_2_ synthesis follows a hydrogen‐free ramping (HFR)‐CVD strategy that we recently developed.^[^ ^26^ ^]^ In this process, pure Ar (50 sccm) was used during the temperature ramping stage, while 5 sccm of H_2_ was only added during the synthesis stage when the temperature reached 850 °C. The conversion of CVD‐synthesized MoSe_2_ and MoS_2_ into Janus ${\mathrm{Mo}}_{\mathrm{Se}}^{S}$ and ${\mathrm{Mo}}_{S}^{\mathrm{Se}}$ follows the H_2_ plasma‐assisted ALS method.^[^ ^6^ ^]^The as‐synthesized TMDs were placed in a tube reactor that is equipped with an inductively coupled plasma system at the upstream, and the distance between the sample and the edge of the plasma coil is optimized to be ≈5–7 cm, depending on the type of starting material (MoSe_2_ or MoS_2_). Sulfur or selenium powder was located near the upstream edge of the plasma coil, providing chalcogen sources for the ALS reaction. During the conversion process, the H radicals in the H_2_ plasma assist the conversion of TMDs to Janus structures. The ALS reaction is kept at room temperature, and the reaction time is 5–15 min.

### STEM Characterization

To prepare STEM specimens, MoSe_2_ flakes were transferred onto a TEM grid (Quantifoil Cu grid). Atomic‐resolution STEM imaging was performed with a probe‐corrected Thermo Fisher Scientific Themis Z G3 60–300 kV S/TEM operated at 200 kV with a beam current of 20–30 pA and 25 mrad convergence angle. All the measurements were conducted at room temperature.

## Conflict of Interest

The authors declare the following competing financial interest(s): HORIBA Scientific is the manufacturer of the equipment used in this study. Collaboration with industry and academia is a part of A.K. job responsibilities. The authors declare no additional conflicts of interest.

## Supporting information



Supporting Information

## Data Availability

The data that support the findings of this study are available from the corresponding author upon reasonable request.
