# Supplementary material for: Synthesis‐Related Nanoscale Defects in Mo‐Based Janus Monolayers Revealed by Cross‐Correlated AFM and TERS Imaging
Source: Small. 2025 Aug 8;21(37):2504742. doi: 10.1002/smll.202504742 (PMC12444857; doi:10.1002/smll.202504742)
Supplement: Supplementary file 1 — Supporting Information [file SMLL-21-2504742-s001.docx]

**Supporting Information for**

**Synthesis-related nanoscale defects in Mo-based Janus monolayers revealed by cross-correlated AFM and TERS imaging**

Tianyi Zhang^1, #^, Andrey Krayev^2, #^, Tilo H. Yang^1^, Nannan Mao^1^, Lauren Hoang^3^, Zhien Wang^1^, Hongwei Liu^1^, Yu-Ren Peng^1,4^, Yunyue Zhu^1^, Xudong Zheng^1^, Eleonora Isotta^5^, Maria E. Kira^6^, Ariete Righi^6^, Marcos A. Pimenta^6^, Yu-Lun Chueh^4^, Eric Pop^3,7^, Andrew J. Mannix^7^, and Jing Kong^1^*

Tianyi Zhang, Tilo H. Yang, Nannan Mao, Zhien Wang, Hongwei Liu, Yu-Ren Peng, Yunyue Zhu, Xudong Zheng, Jing Kong

Department of Electrical Engineering and Computer Science, Massachusetts Institute of Technology, Cambridge, Massachusetts 02139, United States
E-mail: [jingkong@mit.edu](mailto:jingkong@mit.edu)

Andrey Krayev

HORIBA Scientific, Novato, California 94949, United States

Lauren Hoang, Eric Pop

Department of Electrical Engineering, Stanford University, Stanford, California 94305, United States

Yu-Ren Peng, Yu-Lun Chueh

Department of Materials Science and Engineering, National Tsing Hua University, Hsinchu, 30013, Taiwan

Eleonora Isotta

Department of Materials Science and Engineering, Northwestern University, 2220 Campus Drive, Evanston, Illinois 60208, United States

Maria E. Kira, Ariete Righi, Marcos A. Pimenta

Departamento de Física, Universidade Federal de Minas Gerais, Av. Antônio Carlos, 6627, Pampulha, Belo Horizonte, 31270-901, Minas Gerais, Brazil

Eric Pop, Andrew J. Mannix

Department of Materials Science & Engineering, Stanford University, Stanford, California 94305, United States


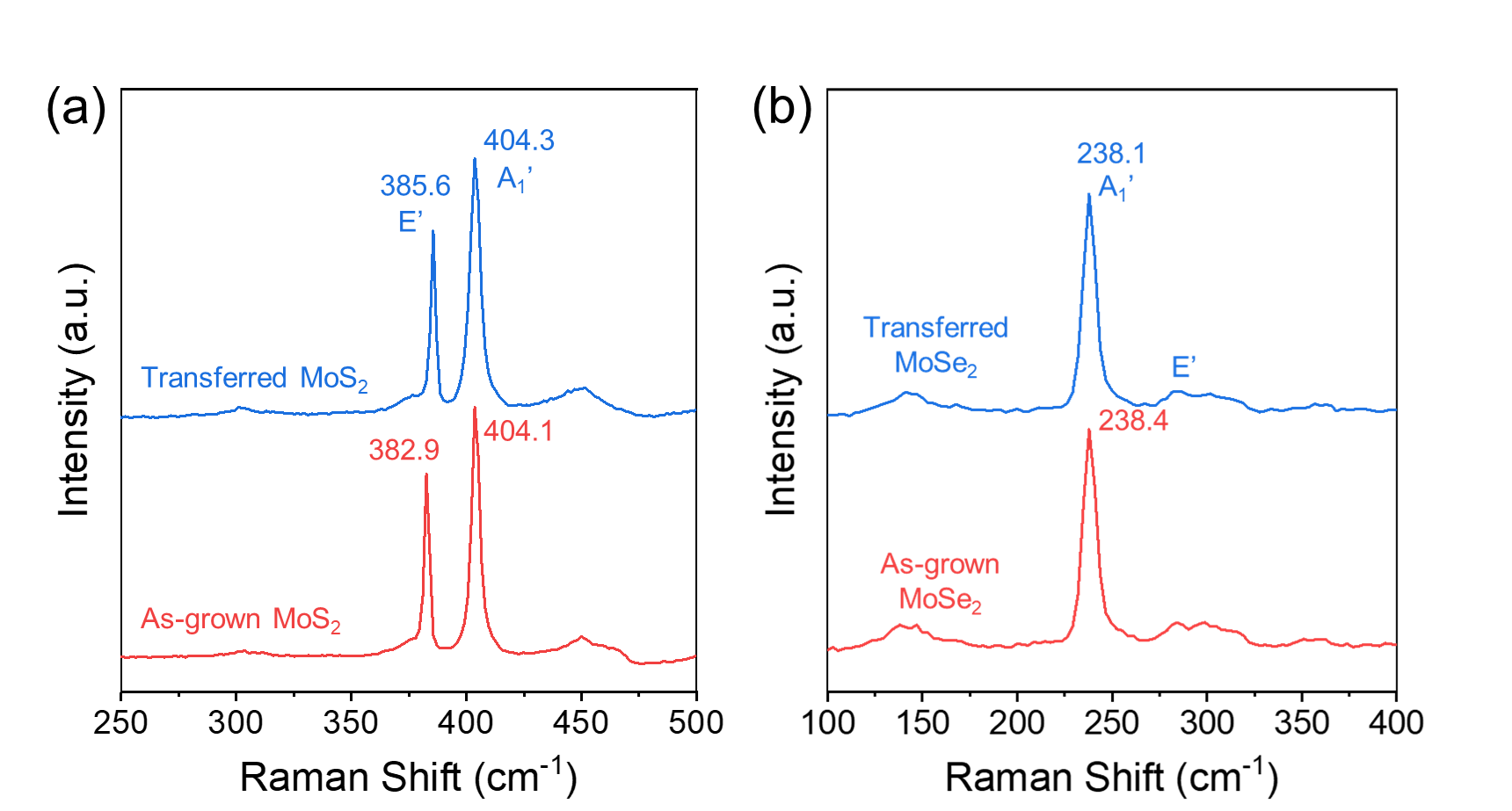


**Figure S1**. **Far-field Raman spectra (532 nm excitation) of chemical vapor deposition (CVD)-grown monolayer MoS_2_ and MoSe_2_ on SiO_2_/Si before and after the transfer process.** For this comparison, the as-grown MoS_2_ and MoSe_2_ were transferred to another piece of SiO_2_/Si, which is identical to the substrate used for their growth. For the Raman spectra of MoS_2_ in (a), after the transfer process which releases the growth-induced strain, the E’ peak blue-shifted by ~2.7 cm^-1^. According to the literature, the E’ Raman mode shift rate as a function of biaxial strain is estimated as -4.48 cm^-1^/%.^[1]^ Thus, we can estimate that the tensile strain in as-grown MoS_2_ is ~0.6%. However, for the Raman spectra of MoSe_2_ in (b), the strain-sensitive E’ mode has a low intensity and cannot be clearly resolved. Since the growth temperature and thermal expansion coefficient (TEC) of monolayer MoSe_2_ are comparable to that of MoS_2_^[2]^, in **Figure 1** of the main manuscript, we estimated the tensile strain in as-grown MoSe_2_ as ~0.6% as well.


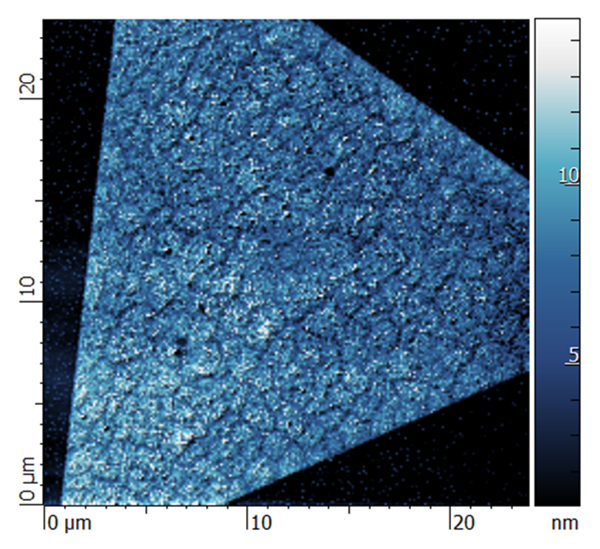

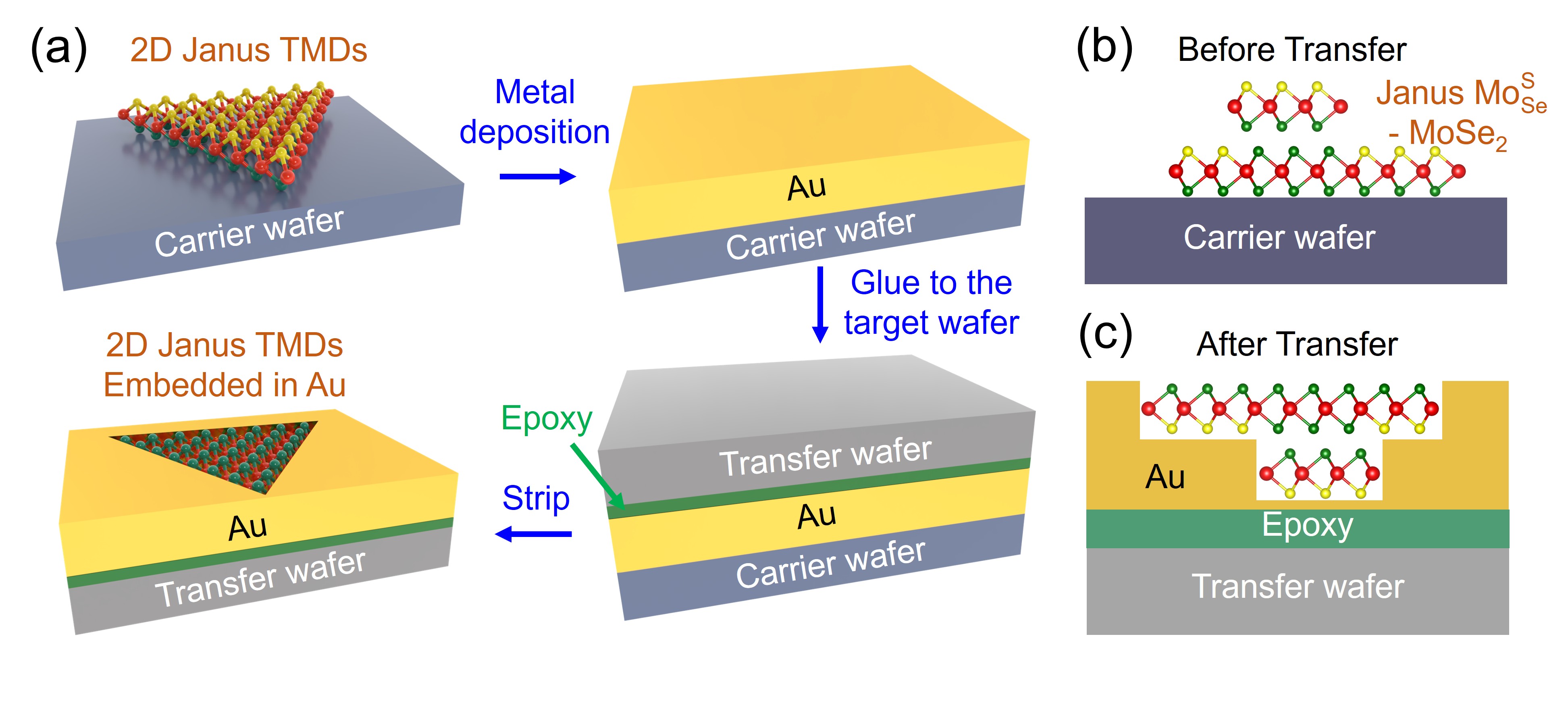
**Figure S2**. **Metal-assisted transfer approach for 2D Janus TMDs.** (a) A step-by-step illustration of the transfer of 2D Janus TMDs to noble metal substrates (e.g., Au, Ag) by using epoxy as a bonding layer. (b-c) Schematics of Janus $\mathrm{Mo}_{\mathrm{Se}}^{S}$-MoSe_2_ vertical heterostructures before and after transfer. The transfer process leads to the heterostructures embedded in Au (or Ag) and vertically flips the crystal orientation.


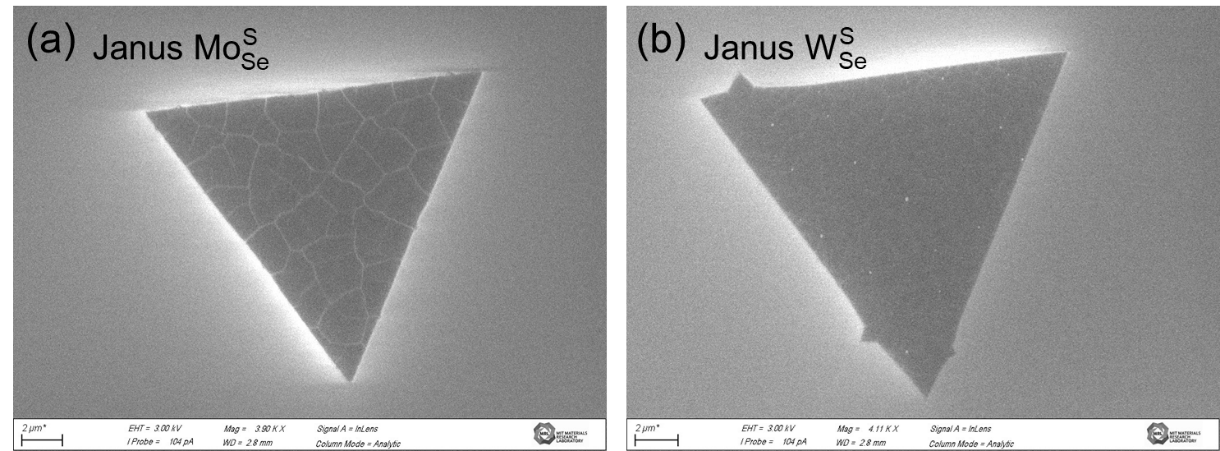
**Figure S3. Atomic force microscopy (AFM) topography image of as-synthesized Janus** $\mathbf{Mo}_{\mathbf{Se}}^{\mathbf{S}}$ **crystal converted from MoSe_2_.** As we can clearly see, the $\mathrm{Mo}_{\mathrm{Se}}^{S}$crystal fragments into small domains.

**Figure S4. Scanning electron microscopy (SEM) images from separate batches of as-synthesized (a) Janus** $\mathbf{Mo}_{\mathbf{Se}}^{\mathbf{S}}$ **and (b) Janus** $\mathbf{W}_{\mathbf{Se}}^{\mathbf{S}}$**.**


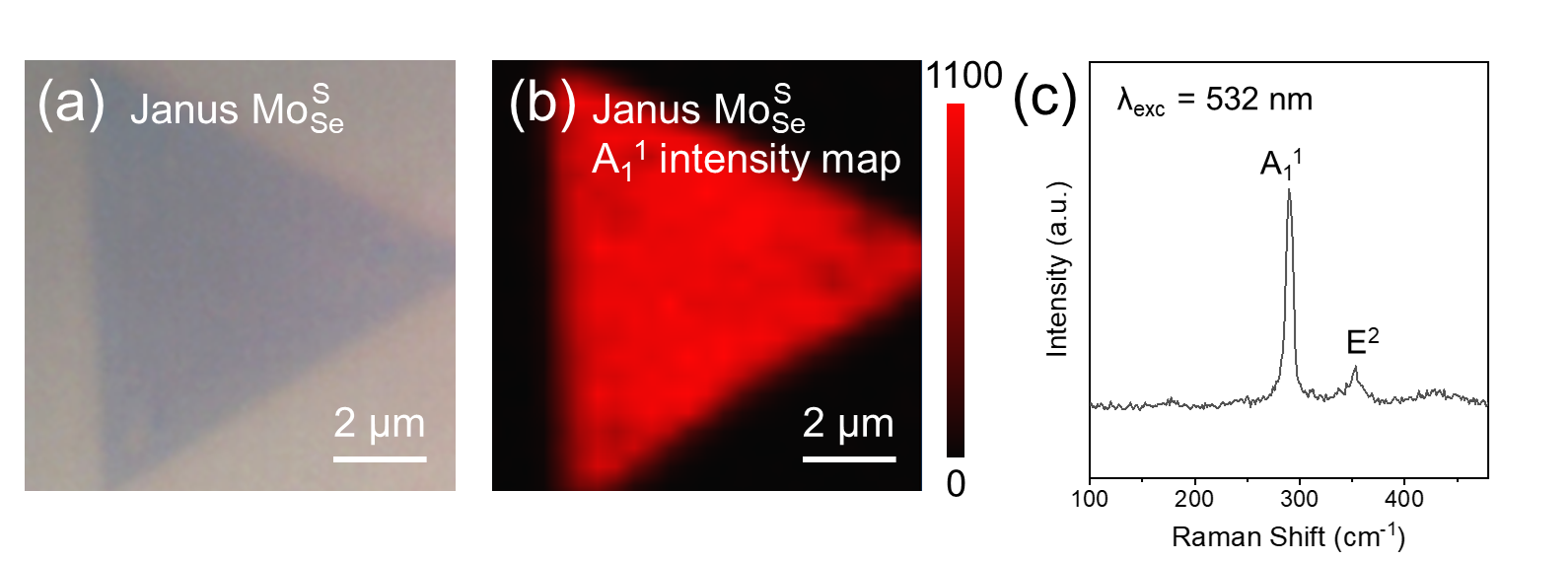
**Figure S5. Far-field optical characterization of Janus** $\mathbf{Mo}_{\mathbf{Se}}^{\mathbf{S}}$**.** (a) Optical image, (b) the corresponding far-field Raman mapping (A_1_^1^ mode intensity), and (c) a typical Raman spectrum of Janus $\mathrm{Mo}_{\mathrm{Se}}^{S}$ extracted from the Raman mapping. The Raman mapping and spectrum were acquired using 532 nm excitation. In (b) and (c), the features of Janus $\mathrm{Mo}_{\mathrm{Se}}^{S}$-MoSe_2_ vertical heterostructures are absent due to diffraction-limited spatial resolution.


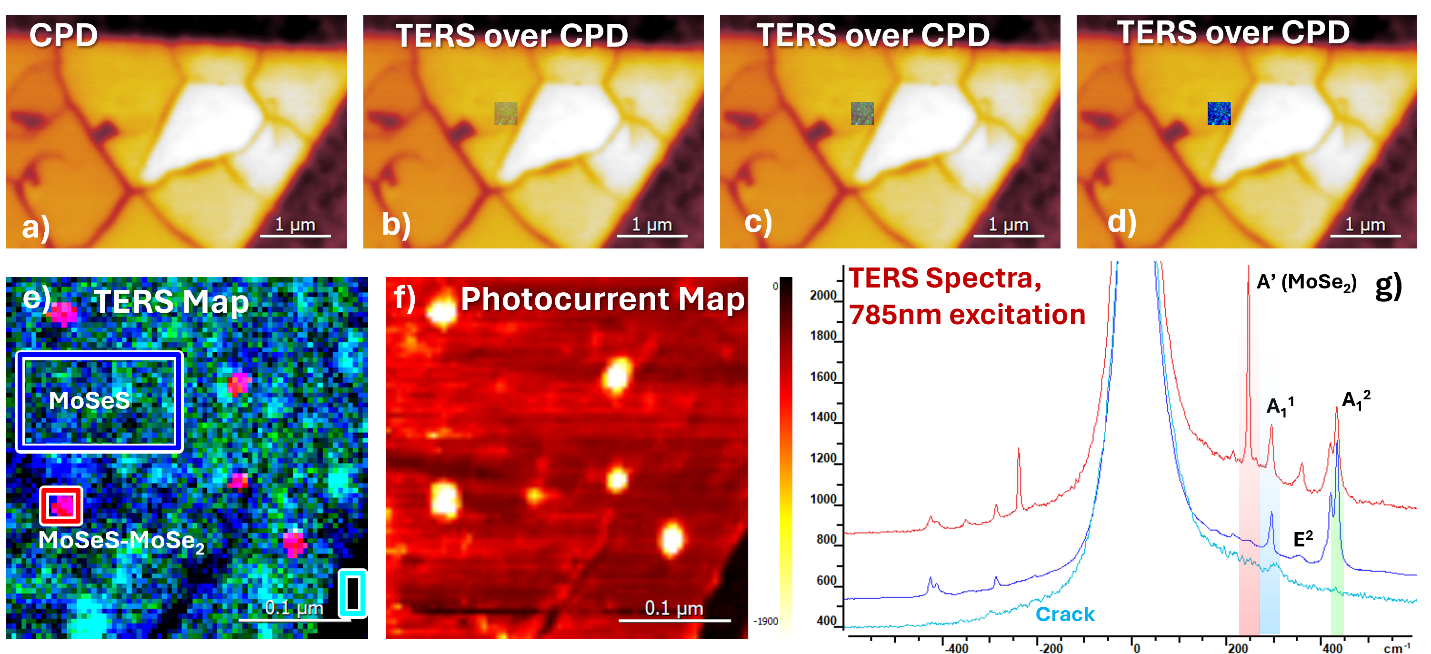


**Figure S6. Cross-correlated contact potential difference (CPD), tip-enhanced Raman spectroscopy (TERS), and photocurrent maps of Janus** $\mathbf{Mo}_{\mathbf{Se}}^{\mathbf{S}}$ **converted from MoSe_2_.** (a) CPD image of the transferred Janus $\mathrm{Mo}_{\mathrm{Se}}^{S}$ flake on the gold substrate. (b-d) TERS maps overlaid over the CPD image of Janus $\mathrm{Mo}_{\mathrm{Se}}^{S}$ with varied transparency. (e) The TERS map showing the intensity distribution of correspondingly highlighted Raman modes in panel (g), namely - MoSe_2_ A_1_’ (red), $\mathrm{Mo}_{\mathrm{Se}}^{S}$ A_1_^2^ (green), and $\mathrm{Mo}_{\mathrm{Se}}^{S}$ A_1_^1^ (blue). (f) Photocurrent map collected concurrently with the TERS map, showing a strong increase in the intensity of the photocurrent over the nanoscale islands of $\mathrm{Mo}_{\mathrm{Se}}^{S}$-MoSe_2_ vertical heterostructures, as should be expected when the photocurrent value is limited by the light absorption. (g) The TERS spectra averaged over the correspondingly colored boxes in panel (e), which represent Janus monolayer $\mathrm{Mo}_{\mathrm{Se}}^{S}$ (blue), $\mathrm{Mo}_{\mathrm{Se}}^{S}$-MoSe_2_ vertical heterostructures (red), and crack (cyan) regions.


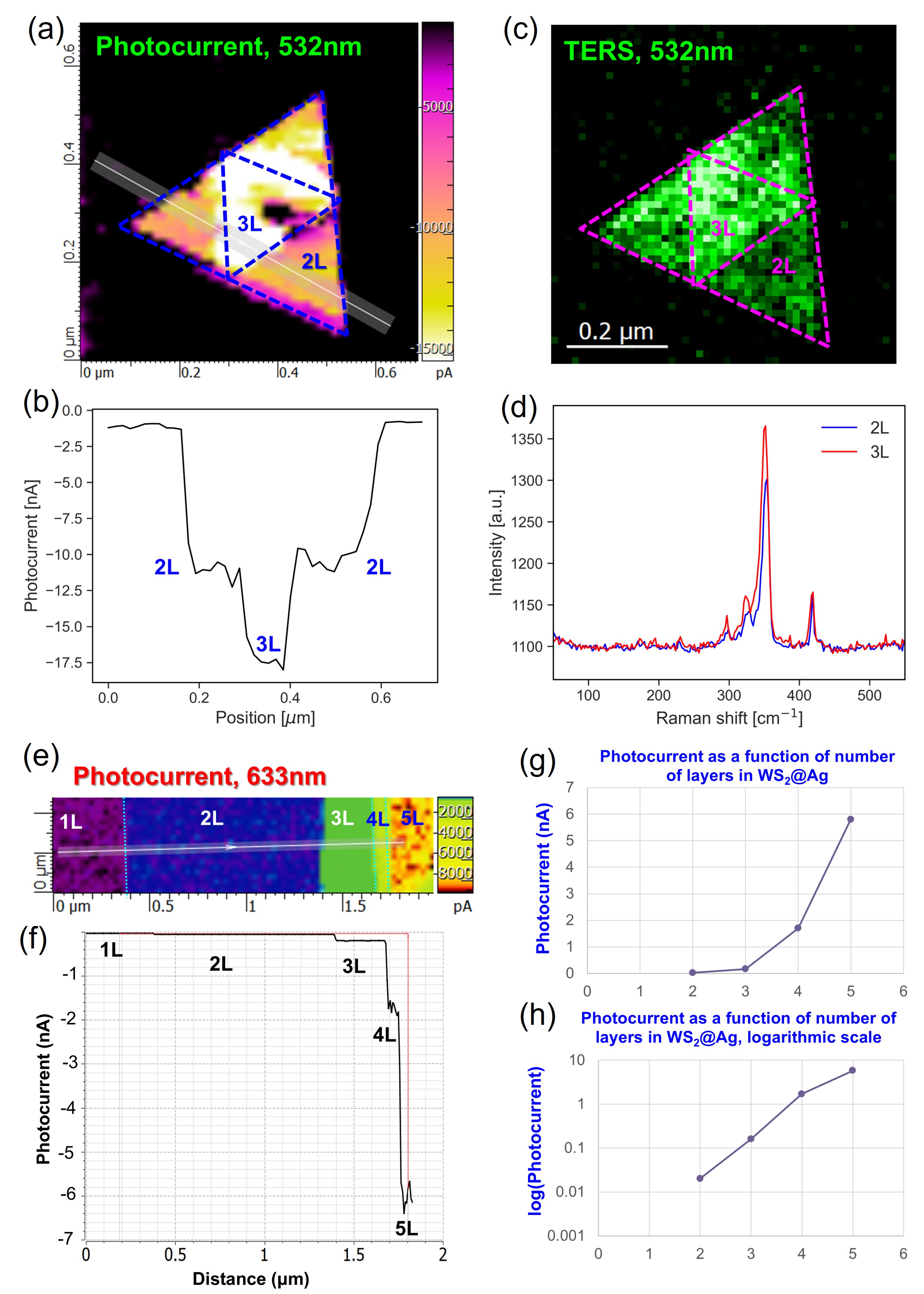
**Figure S7. Photocurrent data as a function of the layer number in WS_2_@Ag for 532 nm and 633 nm excitation.** Photocurrent (a-b) and TERS (c-d) collected with excitation of 532 nm on a multilayer WS_2_@Ag flake, and photocurrent data collected with 633 nm excitation (e-h) on another multilayer flake. As visible, the photocurrent increases with the layer number, which is attributed to the larger absorption possible with more layers, at least within the low layer number limit. The increase in photocurrent with layer number depends on the excitation energy too, and shows an almost exponential trend under illumination close to the A exciton of WS_2_ (see e-h collected with 633 nm excitation wavelength).


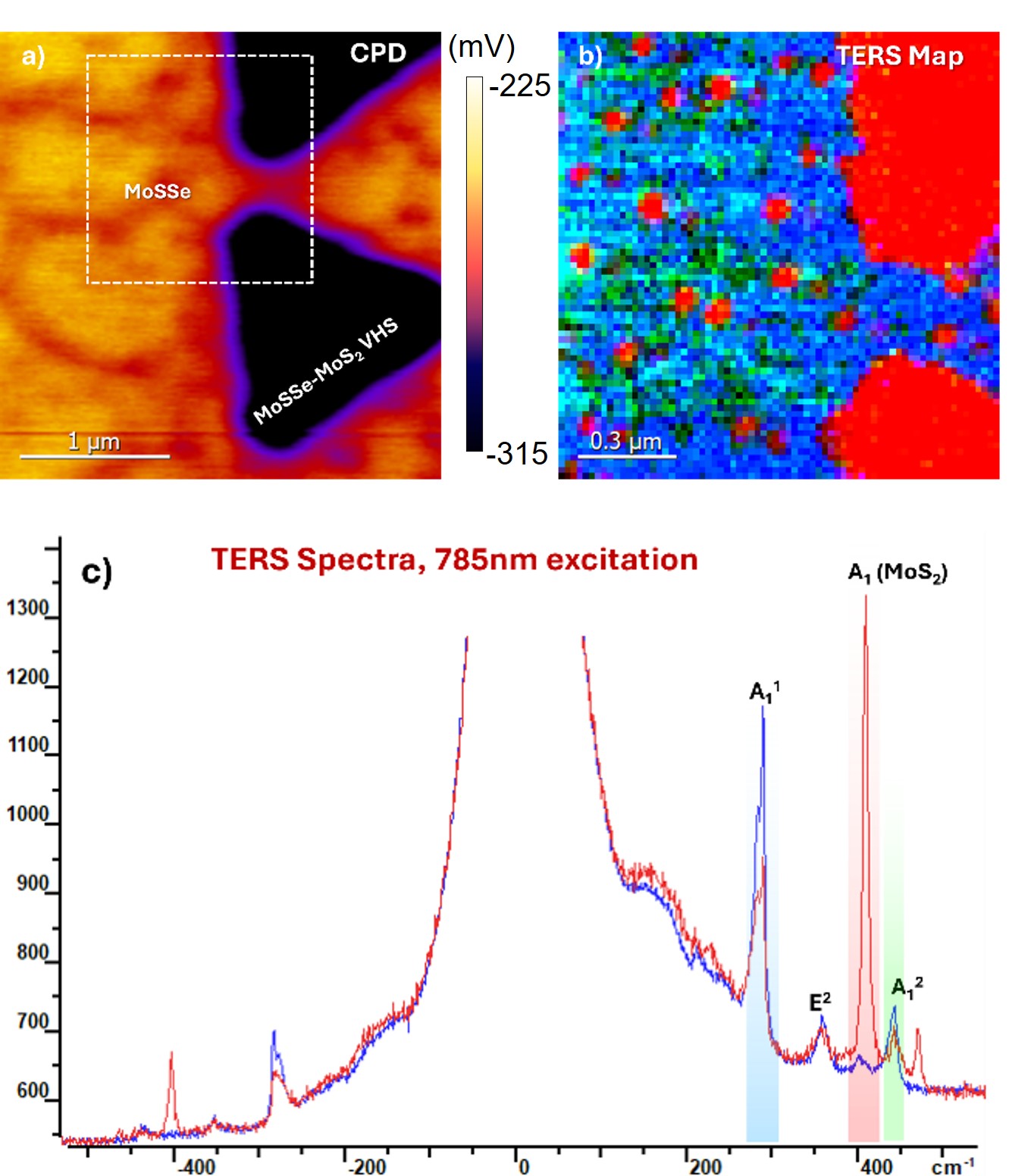

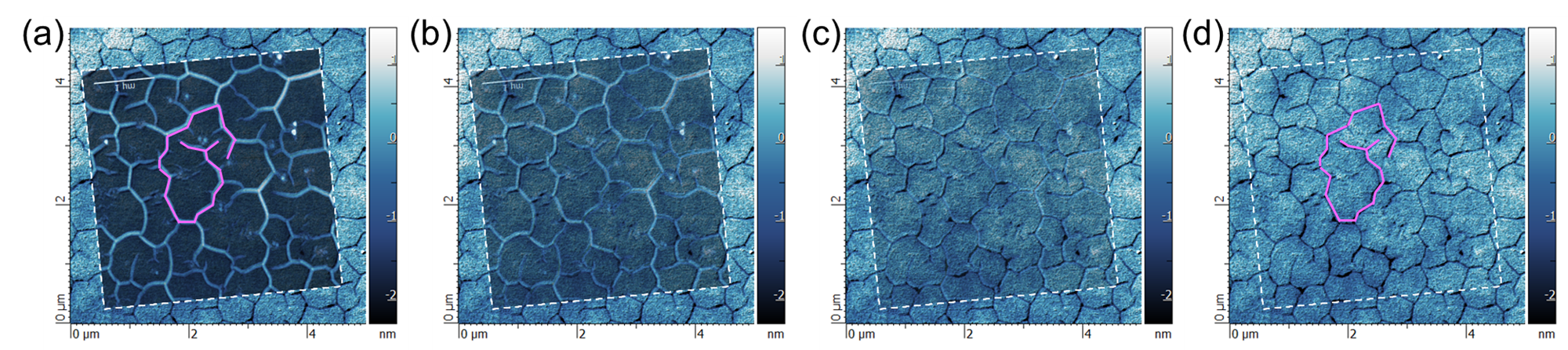
**Figure S8. Superimposed AFM topography images (with varied transparency) of as-grown** **Janus** $\mathbf{Mo}_{\mathbf{S}}^{\mathbf{Se}}$ **on SiO_2_/Si (flipped image) and transferred Janus** $\mathbf{Mo}_{\mathbf{S}}^{\mathbf{Se}}$ **on silver.** We clearly see that the features on as-grown and transferred Janus $\mathrm{Mo}_{S}^{\mathrm{Se}}$ display a perfect match, confirming that the two AFM images were obtained in the same area. Additionally, the wrinkles in the as-grown crystal appears to be “cracks”, but actually, they are inverted wrinkles after the silver-assisted transfer.

**Figure S9. CPD and TERS characterization of microscale and nanoscale islands of Janus** $\mathbf{Mo}_{\mathbf{S}}^{\mathbf{Se}}$**-MoS_2_ converted from MoS_2_.** (a) CPD image of Janus $\mathrm{Mo}_{S}^{\mathrm{Se}}$ transferred onto silver, which captures over 1 µm sized islands of Janus $\mathrm{Mo}_{\mathrm{Se}}^{S}$-MoSe_2_ vertical heterostructures. The area within the white dotted square was imaged using TERS. (b) The TERS map showing the intensity distribution of correspondingly highlighted Raman modes in panel (c), namely - MoS_2_ A_1_’ (red), $\mathrm{Mo}_{S}^{\mathrm{Se}}$ A_1_^2^ (green), and $\mathrm{Mo}_{S}^{\mathrm{Se}}$ A_1_^1^ (blue). Nanoscale islands of Janus $\mathrm{Mo}_{\mathrm{Se}}^{S}$-MoSe_2_, which are undetected by CPD in (a), are resolved by TERS mapping. (c) TERS spectra of Janus monolayer $\mathrm{Mo}_{S}^{\mathrm{Se}}$ (blue) and $\mathrm{Mo}_{S}^{\mathrm{Se}}$-MoS_2_ vertical heterostructures (red).


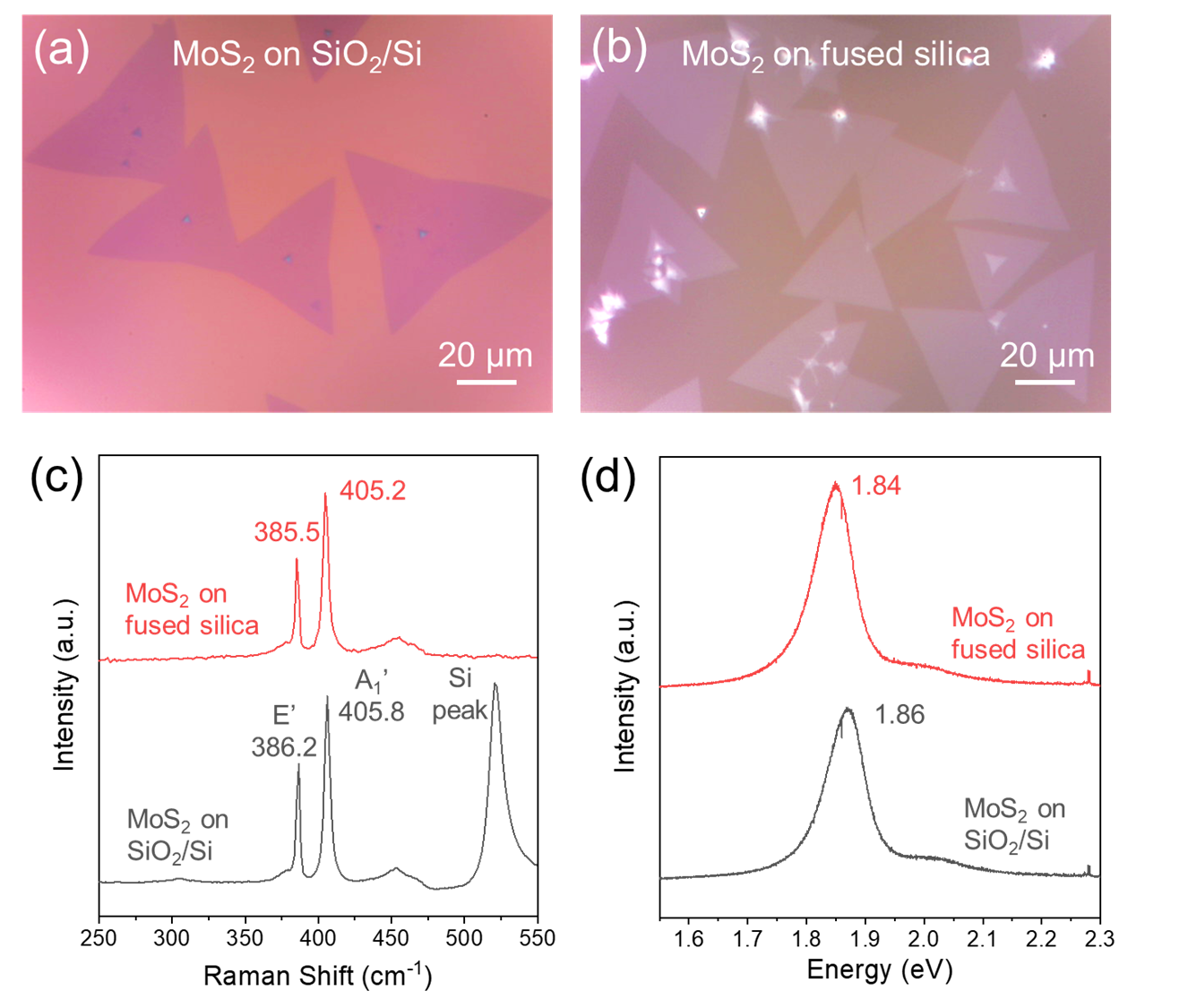
**Figure S10. A comparison between monolayer MoS_2_ grown on SiO_2_/Si and fused silica.** (a) Optical image of as-grown MoS_2_ flakes on SiO_2_/Si. (b) Optical image of as-grown MoS_2_ flakes on fused silica. (c) Normalized Raman spectra and (d) Photoluminescence (PL) of MoS_2_ grown on SiO_2_/Si and fused silica. Red shifts in MoS_2_ E’ Raman mode and PL emission energy are observed in CVD-synthesized monolayer MoS_2_ on fused silica, which indicates a higher amount of tensile strain than that of MoS_2_ grown on SiO_2_/Si.


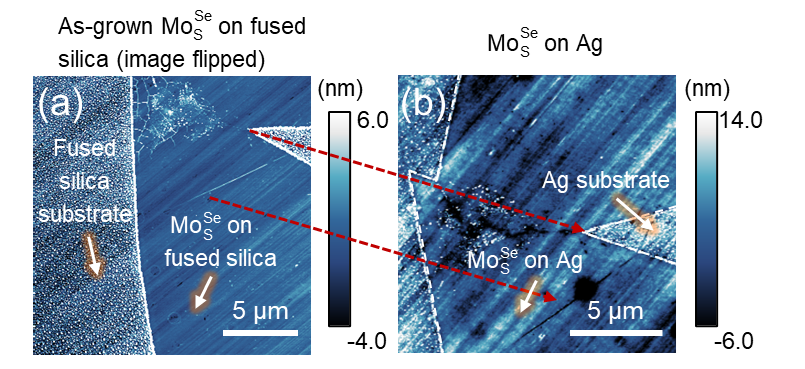


**Figure S11.** **AFM topography images of as-grown** **Janus** $\mathbf{Mo}_{\mathbf{S}}^{\mathbf{Se}}$ **on fused silica (image is flipped to compare with the same region of** $\mathbf{Mo}_{\mathbf{S}}^{\mathbf{Se}}$ **on Ag) and transferred Janus** $\mathbf{Mo}_{\mathbf{S}}^{\mathbf{Se}}$ **on silver.** The morphological characteristics on the Janus $\mathrm{Mo}_{S}^{\mathrm{Se}}$ sample (marked by the red arrows) confirm that the same flake was measured before and after the transfer process. The edges of $\mathrm{Mo}_{S}^{\mathrm{Se}}$ in (b) are outlined by white dashed lines to differentiate $\mathrm{Mo}_{S}^{\mathrm{Se}}$ and bare silver substrate regions.


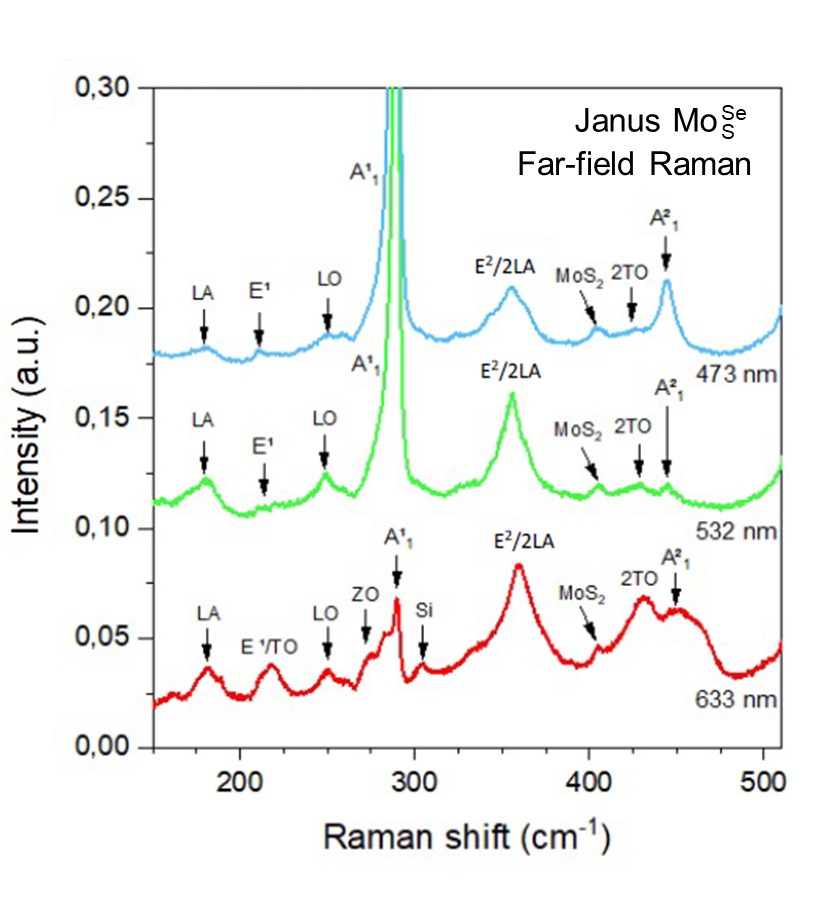
**Figure S12.** **Excitation wavelength-dependent far-field Raman spectra of as-grown Janus** $\mathbf{Mo}_{\mathbf{S}}^{\mathbf{Se}}$ **on SiO_2_/Si.** The relative intensity of different Raman modes (such as A_1_^1^ and A_1_^2^ modes) varies with excitation wavelengths.

**References**

[1] H. Li, A. W. Contryman, X. Qian, S. M. Ardakani, Y. Gong, X. Wang, J. M. Weisse, C. H. Lee, J. Zhao, P. M. Ajayan, J. Li, H. C. Manoharan, X. Zheng, *Nature Communications* **2015**, 6, 7381.

[2] a)L. Zhang, Z. Lu, Y. Song, L. Zhao, B. Bhatia, K. R. Bagnall, E. N. Wang, *Nano Letters* **2019**, 19, 4745; b)Y. Zhong, L. Zhang, J.-H. Park, S. Cruz, L. Li, L. Guo, J. Kong, E. N. Wang, *Science Advances* **2022**, 8, eabo3783.
